# Survival outcomes of low-risk papillary thyroid carcinoma at different risk levels: a corollary for active surveillance

**DOI:** 10.3389/fendo.2023.1235006

**Published:** 2023-09-07

**Authors:** Wu Ding, Guodong Ruan, Yingli Lin, Jianming Zhu, Zhian Li, Dengfeng Ye

**Affiliations:** ^1^ Department of Oncological Surgery, Shaoxing Second Hospital, Shaoxing, China; ^2^ Department of Clinical Medicine, Shaoxing University School of Medicine, Shaoxing, China; ^3^ Department of Early Childhood Education, Shaoxing Vocational & Technical College, Shaoxing, China

**Keywords:** papillary thyroid carcinoma, low-risk, active surveillance, IPTW, PSM

## Abstract

**Background:**

This study aims to compare the outcomes of active surveillance (AS) in low-risk papillary thyroid carcinoma (PTC) patients with different tumor sizes and lymph node metastasis status, in order to establish appropriate management strategies. By analyzing these results, this study provides valuable insights for the effective management of such patients, addressing the issues and challenges associated with AS in practical clinical practice.

**Methods:**

The study utilized the SEER database supported by the National Cancer Institute of the United States, extracting data of PTC diagnosed between 2000 and 2015. Statistical analyses were conducted using inverse probability weighting (IPTW) and propensity score matching (PSM), including Kaplan-Meier survival curves and Cox regression models, to evaluate the impact of different tumor sizes and lymph node metastasis status on thyroid cancer-specific survival (TCSS).

**Results:**

A total of 57,000 PTC patients were included, with most covariates having standardized mean differences below 10% after IPTW and PSM adjustments. The TCSS of PTC with a diameter smaller than 13mm is significantly better than that of tumors with a diameter larger than 13mm, regardless of the presence of lymph node metastasis. Among PTC cases with a diameter smaller than 13mm, the TCSS of patients is similar, regardless of the presence of lymph node metastasis. However, in PTC cases with a diameter larger than 13mm, the presence of lateral neck lymph node metastasis (N1b stage) significantly impacts the TCSS, although the absolute impact on TCSS rate is minimal.

**Conclusion:**

The treatment strategy of AS is safe for patients with T1a stage papillary thyroid microcarcinoma (PTMC). However, for patients with T1b stage, if the tumor diameter exceeds 13mm or there is lymph node metastasis in the lateral neck region, the TCSS will be significantly affected. Nevertheless, the absolute impact on survival is relatively small.

## Introduction

In recent decades, the global incidence of thyroid cancer, particularly papillary thyroid microcarcinoma (PTMC), has significantly increased. This rise can be attributed to factors like widespread ultrasound use, technological advancements, and population screening programs (1–4). Although the incidence of PTMC continues to rise, mortality rates have remained relatively stable (5). Consequently, there has been a reevaluation of the traditional approach of immediate thyroid surgery for PTMC (6), leading to the emergence of active surveillance (AS) as a conservative management strategy for low-risk PTMC cases (7).

During AS, disease progression is defined by tumor enlargement or the development of lymph node or distant metastasis, with the latter being rare. Notably, long-term prospective trials conducted by Kuma Hospital and Cancer Institute Hospital, two prominent Japanese institutions, have evaluated the safety and effectiveness of AS for asymptomatic PTMC patients with T1aN0M0 status. Kuma Hospital’s experience over a 10-year follow-up revealed an 8.0% incidence of tumor growth and a 3.8% incidence of cervical lymph node spread (7). Similarly, the Cancer Institute Hospital reported a 7% rate of tumor size enlargement and a 1% occurrence of new lymph node metastasis, without extrathyroidal invasion or distant metastasis (8). Based on these positive outcomes, the Japanese Clinical Guidelines for thyroid tumor treatment, published in 2010, approved AS as a management option for asymptomatic PTMC patients, making it the first country to do so (9). The American Thyroid Association (ATA) also revised its guidelines to support AS as a suitable alternative to immediate surgery for selected patients with very low-risk tumors (10).

Despite the recommendation of AS, concerns persist among healthcare providers regarding its implementation in real-world practice. Some scholars have observed aggressive central lymph node metastasis even in small-sized lesions (11, 12), which creates a paradox of “low-risk” classification alongside a high prevalence of lymph node metastasis. However, it is worth noting that tumor enlargement or lymph node metastasis may not necessarily increase the recurrence rate or mortality of the disease. Additionally, there is a growing belief that patients with T1bN0M0 status are also suitable candidates for AS (13). To establish an appropriate management strategy for cT1aN0M0 or cT1bN0M0 papillary thyroid carcinoma (PTC) patients, this study aims to compare AS outcomes among low-risk patients with varying tumor sizes and lymph node metastasis. Analyzing these outcomes will provide valuable insights for the effective management of such patients, addressing concerns and challenges associated with the implementation of AS in real-world clinical practice.

## Methods

### Data source

The SEER database, supported by the National Cancer Institute, collects case data from population-based cancer registries covering approximately 34.6 percent of the U.S. population. It contains de-identified information on patient demographics, primary tumor site, tumor morphology, stage at diagnosis, and follow-up vital status. For this study, we utilized data on papillary thyroid cancer diagnosed between 2000 and 2015, obtained from the November 2022 submission of SEER. The study was reviewed and approved by the institutional review board at Shaoxing Second Hospital, and the data were deidentified, thus patient consent was not required.

### Patient selection

We employed SEER*Stat 8.4.1 software to extract relevant information, including patient identification, year of diagnosis (2000-2003, 2004-2007, 2008-2011, 2012-2015), patient age (<55, ≥55), race/ethnicity (white, black, others), marital status (married, single, divorced, separated or widowed), gender (male, female), geographic region (metropolitan, nonmetropolitan), months from diagnosis to treatment (<6, 6-12, 12-24), histological type, tumor size (≤20 mm), N stage (N0, N1a, N1b), distant metastases, multifocality, surgery type (none, less than lobectomy, lobectomy, subtotal or near or total thyroidectomy), lymph node dissection (yes or none), chemotherapy (yes or none), and radiation therapy (none, radioisotopes (RAI), external radiotherapy, combined radioisotopes and external radiotherapy). All variables were categorized as outlined in **Table 1**. Only patients with biopsy-proven well-differentiated PTC were included. ICD-03 histologies comprised 8050, 8260, 8340–8344, 8350, and 8450–8460. To select only T1N0M0 low-risk PTC cases, patients with tumors larger than 20 mm, carcinoma in situ, extrathyroidal extension, incomplete lymph node status, distant metastases, or PTC diagnosis at autopsy were excluded. Patients who received external radiotherapy or chemotherapy or had incomplete surgical information were also excluded to enhance data accuracy and minimize potential confounding effects of subtherapeutic regimens.

**Table 1:** Baseline Demographic and Tumor Characteristics of PTC Patients with Different Lymph Node Status and Tumor Sizes in the SEER Database.

Characteristics	N0						N1a						N1b					
	1-4mm N=15362	5-7mm N=9156	8-10mm N=8,630	11-13mm N=5,786	14-16mm N=5,517	17-20mm N=5,572	1-4mm N=351	5-7mm N=620	8-10mm N=999	11-13mm N=944	14-16mm N=931	17-20mm N=1,056	1-4mm N=245	5-7mm N=324	8-10mm N=396	11-13mm N=363	14-16mm N=374	17-20mm N=374
Year of diagnosis																		
2000-2003	1954 (12.7)	930 (10.2)	970 (11.2)	642 (11.1)	735 (13.3)	876 (15.7)	64 (18.2)	93 (15.0)	104 (10.4)	67 (7.1)	94 (10.1)	130 (12.3)	10 (4.1)	11 (3.4)	17 (4.3)	19 (5.2)	19 (5.1)	45 (12.0)
2004-2007	3249 (21.1)	1905 (20.8)	1760 (20.4)	1190 (20.6)	1293 (23.4)	1342 (24.1)	54 (15.4)	97 (15.6)	126 (12.6)	149 (15.8)	131 (14.1)	204 (19.3)	53 (21.6)	76 (23.5)	91 (23.0)	59 (16.3)	72 (19.3)	78 (20.9)
2008-2011	4781 (31.1)	2893 (31.6)	2731 (31.6)	1790 (30.9)	1653 (30.0)	1614 (29.0)	118 (33.6)	179 (28.9)	327 (32.7)	313 (33.2)	301 (32.3)	311 (29.5)	69 (28.2)	110 (34.0)	119 (30.1)	122 (33.6)	135 (36.1)	111 (29.7)
2012-2015	5378 (35.0)	3428 (37.4)	3169 (36.7)	2164 (37.4)	1836 (33.3)	1740 (31.2)	115 (32.8)	251 (40.5)	442 (44.2)	415 (44.0)	405 (43.5)	411 (38.9)	113 (46.1)	127 (39.2)	169 (42.7)	163 (44.9)	148 (39.6)	140 (37.4)
Age (years)																		
<55	8891 (57.9)	5832 (63.7)	5782 (67.0)	3981 (68.8)	3849 (69.8)	3977 (71.4)	250 (71.2)	480 (77.4)	785 (78.6)	786 (83.3)	745 (80.0)	865 (81.9)	157 (64.1)	246 (75.9)	311 (78.5)	290 (79.9)	303 (81.0)	294 (78.6)
≥55	6471 (42.1)	3324 (36.3)	2848 (33.0)	1805 (31.2)	1668 (30.2)	1595 (28.6)	101 (28.8)	140 (22.6)	214 (21.4)	158 (16.7)	186 (20.0)	191 (18.1)	88 (35.9)	78 (24.1)	85 (21.5)	73 (20.1)	71 (19.0)	80 (21.4)
Gender																		
Male	2421 (15.8)	1470 (16.1)	1411 (16.3)	941 (16.3)	965 (17.5)	1015 (18.2)	107 (30.5)	173 (27.9)	224 (22.4)	195 (20.7)	200 (21.5)	252 (23.9)	105 (42.9)	101 (31.2)	134 (33.8)	104 (28.7)	111 (29.7)	109 (29.1)
Female	12941 (84.2)	7686 (83.9)	7219 (83.7)	4845 (83.7)	4552 (82.5)	4557 (81.8)	244 (69.5)	447 (72.1)	775 (77.6)	749 (79.3)	731 (78.5)	804 (76.1)	140 (57.1)	223 (68.8)	262 (66.2)	259 (71.3)	263 (70.3)	265 (70.9)
Race																		
White	12797 (83.3)	7552 (82.5)	7187 (83.3)	4767 (82.4)	4573 (82.9)	4609 (82.7)	292 (83.2)	541 (87.3)	868 (86.9)	756 (80.1)	777 (83.5)	908 (86.0)	214 (87.3)	276 (85.2)	329 (83.1)	307 (84.6)	321 (85.8)	303 (81.0)
Black	1161 (7.6)	616 (6.7)	483 (5.6)	328 (5.7)	310 (5.6)	347 (6.2)	14 (4.0)	15 (2.4)	19 (1.9)	22 (2.3)	28 (3.0)	25 (2.4)	7 (2.9)	13 (4.0)	13 (3.3)	10 (2.8)	11 (2.9)	13 (3.5)
Other	1263 (8.2)	906 (9.9)	902 (10.5)	639 (11.0)	585 (10.6)	577 (10.4)	38 (10.8)	58 (9.4)	102 (10.2)	154 (16.3)	119 (12.8)	117 (11.1)	22 (9.0)	34 (10.5)	49 (12.4)	45 (12.4)	40 (10.7)	56 (15.0)
Unknown	141 (0.9)	82 (0.9)	58 (0.7)	52 (0.9)	49 (0.9)	39 (0.7)	7 (2.0)	6 (1.0)	10 (1.0)	12 (1.3)	7 (0.8)	6 (0.6)	2 (0.8)	1 (0.3)	5 (1.3)	1 (0.3)	2 (0.5)	2 (0.5)
Marital Status																		
single	2371 (15.4)	1524 (16.6)	1581 (18.3)	1093 (18.9)	1079 (19.6)	1092 (19.6)	67 (19.1)	147 (23.7)	247 (24.7)	258 (27.3)	254 (27.3)	263 (24.9)	57 (23.3)	87 (26.9)	113 (28.5)	95 (26.2)	110 (29.4)	107 (28.6)
Married	9942 (64.7)	5985 (65.4)	5658 (65.6)	3735 (64.6)	3595 (65.2)	3629 (65.1)	227 (64.7)	383 (61.8)	639 (64.0)	585 (62.0)	549 (59.0)	661 (62.6)	147 (60.0)	199 (61.4)	230 (58.1)	221 (60.9)	212 (56.7)	224 (59.9)
Widowed/divorced	2225 (14.5)	1246 (13.6)	1000 (11.6)	729 (12.6)	617 (11.2)	624 (11.2)	41 (11.7)	67 (10.8)	73 (7.3)	60 (6.4)	84 (9.0)	90 (8.5)	30 (12.2)	28 (8.6)	38 (9.6)	38 (10.5)	35 (9.4)	31 (8.3)
Unknown	824 (5.4)	401 (4.4)	391 (4.5)	229 (4.0)	226 (4.1)	227 (4.1)	16 (4.6)	23 (3.7)	40 (4.0)	41 (4.3)	44 (4.7)	42 (4.0)	11 (4.5)	10 (3.1)	15 (3.8)	9 (2.5)	17 (4.5)	12 (3.2)
Rural																		
metropolitan	13580 (88.4)	8212 (89.7)	7805 (90.4)	5257 (90.9)	4979 (90.2)	5049 (90.6)	331 (94.3)	581 (93.7)	935 (93.6)	875 (92.7)	871 (93.6)	985 (93.3)	219 (89.4)	304 (93.8)	366 (92.4)	342 (94.2)	355 (94.9)	352 (94.1)
Nonmetropolitan	1782 (11.6)	944 (10.3)	825 (9.6)	529 (9.1)	538 (9.8)	523 (9.4)	20 (5.7)	39 (6.3)	64 (6.4)	69 (7.3)	60 (6.4)	71 (6.7)	26 (10.6)	20 (6.2)	30 (7.6)	21 (5.8)	19 (5.1)	22 (5.9)
Number of lesions																		
Unifocality	12024 (78.3)	6147 (67.1)	5432 (62.9)	3543 (61.2)	3485 (63.2)	3612 (64.8)	199 (56.7)	309 (49.8)	531 (53.2)	499 (52.9)	512 (55.0)	593 (56.2)	128 (52.2)	130 (40.1)	167 (42.2)	167 (46.0)	167 (44.7)	207 (55.3)
Multifocality	3338 (21.7)	3009 (32.9)	3198 (37.1)	2243 (38.8)	2032 (36.8)	1960 (35.2)	152 (43.3)	311 (50.2)	468 (46.8)	445 (47.1)	419 (45.0)	463 (43.8)	117 (47.8)	194 (59.9)	229 (57.8)	196 (54.0)	207 (55.3)	167 (44.7)
Thyroidectomy																		
None	4 (0.0)	8 (0.1)	6 (0.1)	11 (0.2)	2 (0.0)	7 (0.1)	1 (0.3)	0 (0.0)	1 (0.1)	0 (0.0)	0 (0.0)	0 (0.0)	0 (0.0)	0 (0.0)	0 (0.0)	2 (0.6)	1 (0.3)	0 (0.0)
Hemithyroidectomy	5479 (35.7)	2147 (23.4)	1331 (15.4)	587 (10.1)	614 (11.1)	616 (11.1)	26 (7.4)	34 (5.5)	35 (3.5)	19 (2.0)	24 (2.6)	30 (2.8)	4 (1.6)	3 (0.9)	4 (1.0)	0 (0.0)	3 (0.8)	7 (1.9)
Total thyroidectomy	9879 (64.3)	7001 (76.5)	7293 (84.5)	5188 (89.7)	4901 (88.8)	4949 (88.8)	324 (92.3)	586 (94.5)	963 (96.4)	925 (98.0)	907 (97.4)	1026 (97.2)	241 (98.4)	321 (99.1)	392 (99.0)	361 (99.4)	370 (98.9)	367 (98.1)
Lymph node dissection																		
None	10888 (70.9)	5818 (63.5)	5049 (58.5)	3203 (55.4)	3206 (58.1)	3306 (59.3)	1 (0.3)	1 (0.2)	3 (0.3)	2 (0.2)	1 (0.1)	3 (0.3)	2 (0.8)	1 (0.3)	7 (1.8)	8 (2.2)	4 (1.1)	8 (2.1)
Yes	4474 (29.1)	3338 (36.5)	3581 (41.5)	2583 (44.6)	2311 (41.9)	2266 (40.7)	350 (99.7)	619 (99.8)	996 (99.7)	942 (99.8)	930 (99.9)	1053 (99.7)	243 (99.2)	323 (99.7)	389 (98.2)	355 (97.8)	370 (98.9)	366 (97.9)
RAI																		
None	13903 (90.5)	7174 (78.4)	5590 (64.8)	2976 (51.4)	2599 (47.1)	2400 (43.1)	121 (34.5)	189 (30.5)	267 (26.7)	255 (27.0)	241 (25.9)	260 (24.6)	68 (27.8)	69 (21.3)	97 (24.5)	71 (19.6)	86 (23.0)	80 (21.4)
Yes	1459 (9.5)	1982 (21.6)	3040 (35.2)	2810 (48.6)	2918 (52.9)	3172 (56.9)	230 (65.5)	431 (69.5)	732 (73.3)	689 (73.0)	690 (74.1)	796 (75.4)	177 (72.2)	255 (78.7)	299 (75.5)	292 (80.4)	288 (77.0)	294 (78.6)
Follow-up time (mean (SD))																		
	125.97 (53.34)	123.46 (51.69)	124.76 (52.19)	124.83 (52.82)	129.80 (54.16)	134.00 (55.67)	125.52 (58.71)	125.95 (56.05)	117.13 (52.43)	115.76 (48.43)	119.07 (51.39)	125.12 (54.51)	111.41 (52.56)	116.70 (48.52)	118.15 (47.05)	114.21 (46.31)	118.99 (47.64)	127.02 (55.76)

RAI, radioactive iodine.

### Statistical analysis

We employed similar statistical analysis approaches as previous studies that examined the benefit of interventions for breast cancer subsets (14, 15). Baseline patient, tumor, and treatment characteristics were compared between different tumor size groups and N stage groups using Pearson’s Chi-square test. To address missing data, we performed multiple imputation using a multivariate logistic regression model, with 10 cycles repeated to produce a final dataset. The imputation model included the following variables: year of diagnosis (2000-2003, 2004-2007, 2008-2011, 2012-2015), patient age (<55, ≥55), race/ethnicity (white, black, others), marital status (married, single, divorced, separated or widowed), gender (male, female), geographic region (metropolitan, nonmetropolitan), months from diagnosis to treatment (<6, 6-12, 12-24), tumor size (≤20 mm), N stage (N0, N1a, N1b), distant metastases, multifocality, surgery type (none, less than lobectomy, lobectomy, subtotal or near or total thyroidectomy), lymph node dissection (yes or none), chemotherapy (yes or none), and radiation therapy (none, radioisotopes (RAI)).

To balance the bias of confounding factors that may affect chemotherapy allocation, we utilized inverse probability of treatment weighting (IPTW) and propensity score matching (PSM) with a 1:1 ratio and a caliper of 0.01 (16, 17). Multivariable logistic regression was performed to generate propensity scores (PS) for all variables, and then weights were calculated and matching was conducted based on the PS. PSM-adjusted Kaplan-Meier curves were analyzed using log-rank tests between different tumor size groups and N stage groups. Subsequently, IPTW-adjusted multivariable Cox regression models were created, and hazard ratios (HR) for thyroid cancer-specific survival (TCSS) between different tumor size groups and N stage groups were recalculated. Subgroup analyses were also conducted following the same procedures.

Furthermore, to evaluate the stability of our findings, we performed a comprehensive set of sensitivity analyses. Firstly, we utilized a proportional subdistribution hazards model to calculate hazard ratios (HR) for different tumor size groups and N stage groups, while adjusting for competing events such as death from other causes (18). Secondly, we repeated the entire analysis employing multiple imputation techniques to address missing data, using the random survival forest methodology.

All P values were calculated from two-sided tests with a significance threshold of 0.05 to evaluate the statistical significance of survival benefit by surgery. All statistical analyses were performed using R software (version 3.6.3).

## Results

### Baseline characteristics of patients

**Figure 1:**
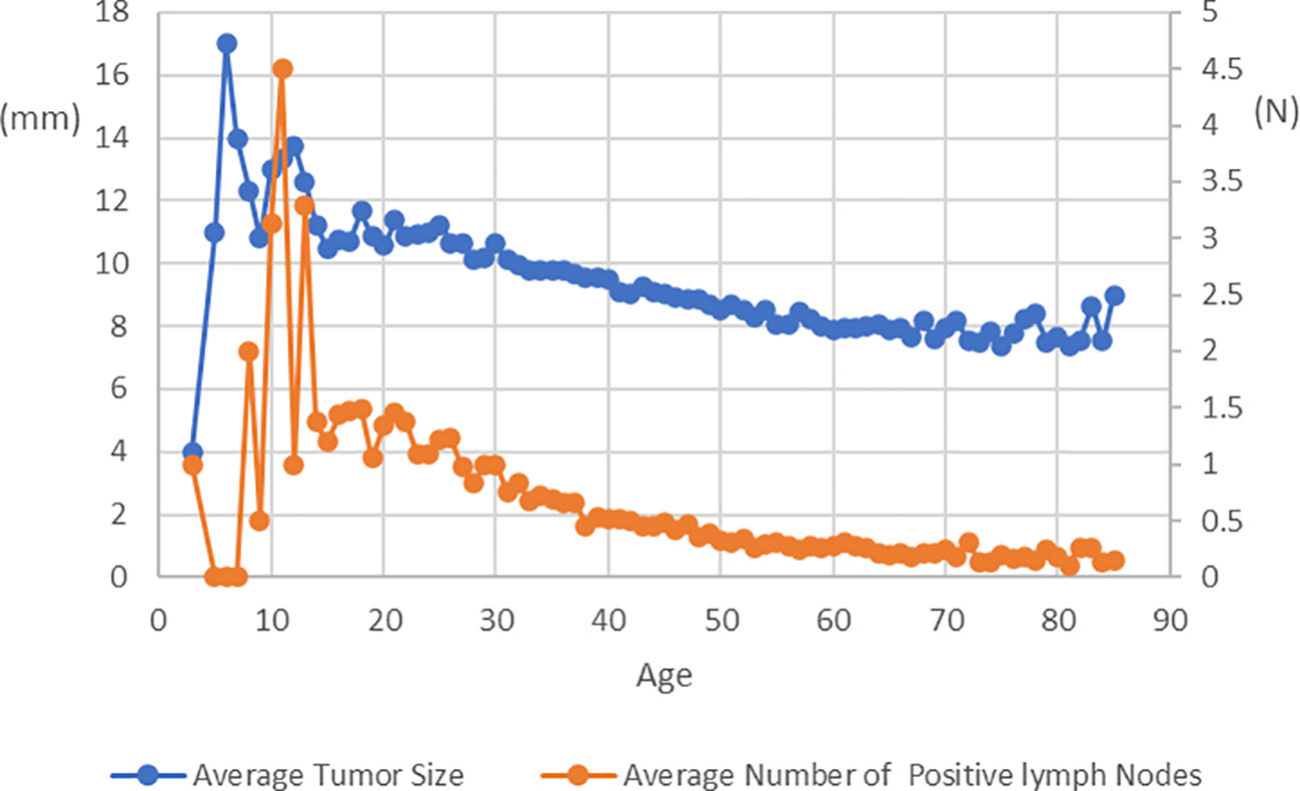
Average Tumor Size and Average Number of Positive lymph Nodes at Diagnosis in Patients with PTC of Different Age.

A total of 57,000 patients with histologically confirmed PTC were included in our study. The patients were categorized into groups based on tumor size and N stage. The average follow-up period was 125.69 months (standard deviation: 53.14). **Table 1** presents the clinicopathological characteristics of the study cohort. To address confounding factors, we applied IPTW and PSM, resulting in standardized mean differences (SMDs) below 10% for most covariates in the IPTW and PSM cohorts, indicating successful mitigation of confounding effects. **Figure 1** illustrates age-related changes in mean tumor size and the average number of positive lymph nodes. The figure demonstrates a consistent decline in both tumor size and positive lymph node numbers after the age of 20, with a stable trend observed around the age of 55.

### Kaplan-Meier survival curves adjusted for PSM

**Figure 2:**
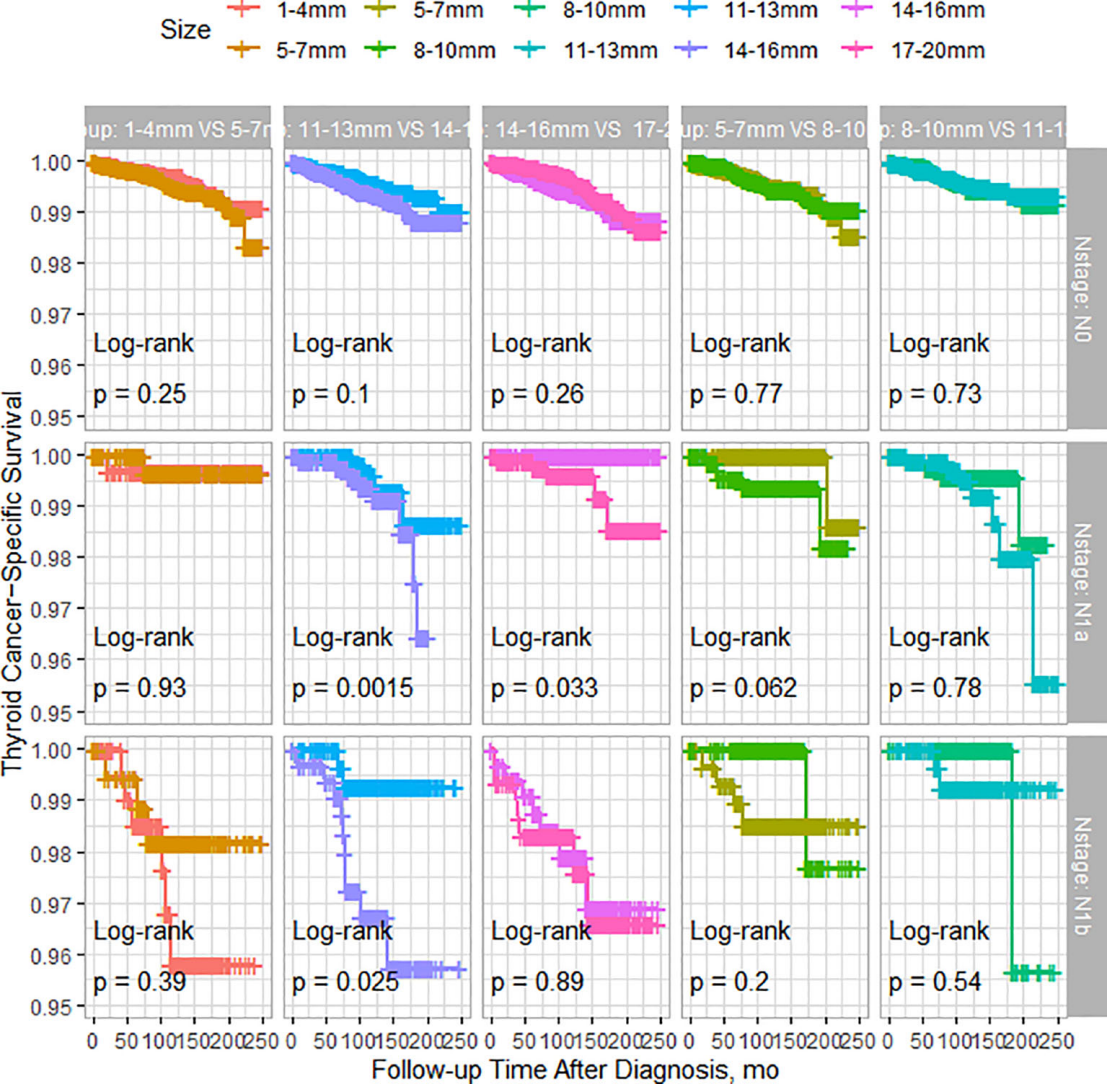
PSM-Adjusted Kaplan-Meier Curves Comparing Thyroid Cancer-Specific Survival Rates of Patients with Different Tumor Sizes: 1-4mm vs. 5-7mm, 5-7mm vs. 8-10mm, 8-10mm vs. 11-13mm, 11-13mm vs. 14-16mm, 14-16mm vs. 17-20mm, Stratified by Lymph Node Status.

**Figure 3:**
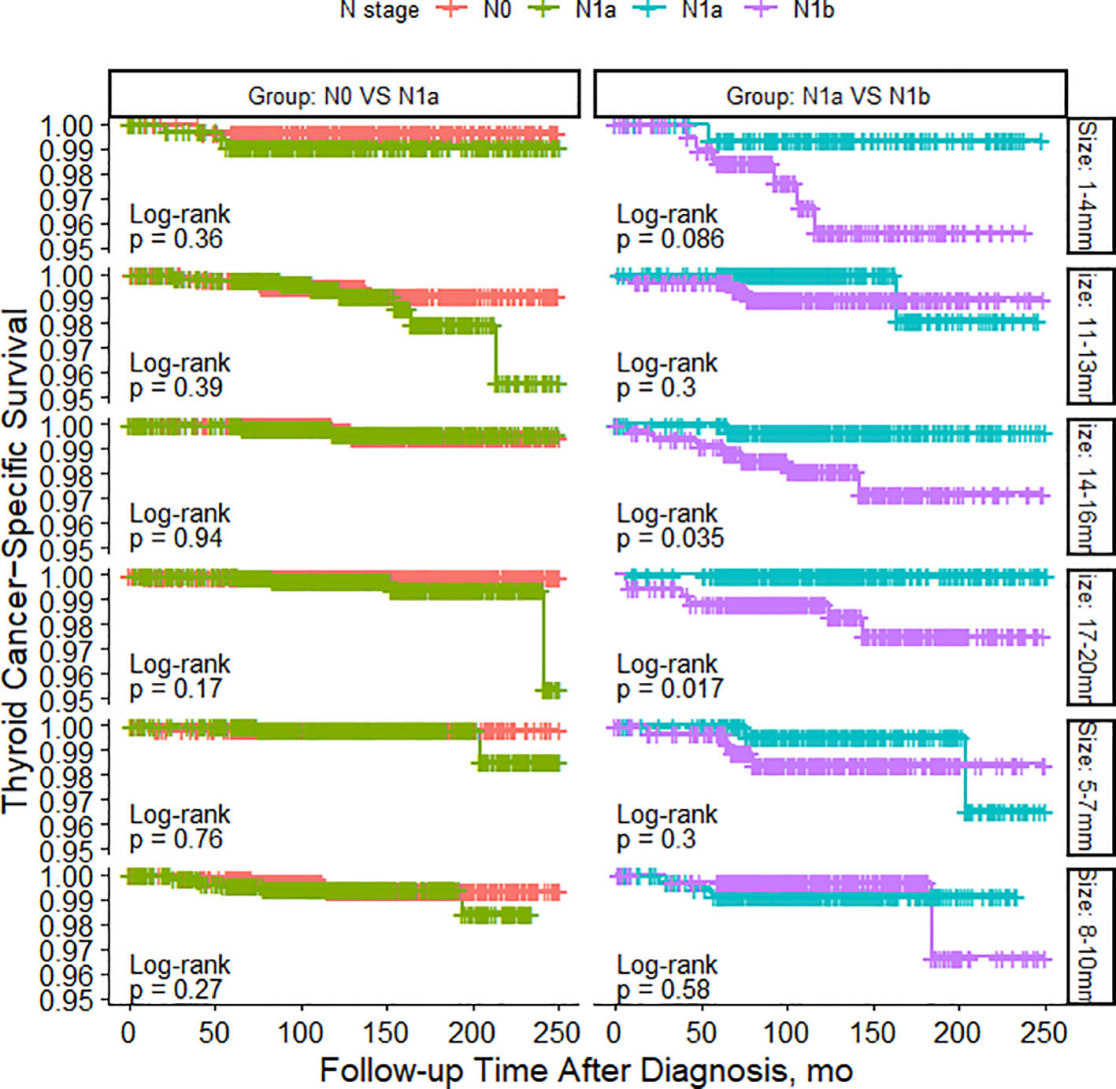
PSM-Adjusted Kaplan-Meier Curves Comparing Thyroid Cancer-Specific Survival Rates of Patients with Different Lymph Node Status: N0 to N1a and N1a to N1b, Stratified by Tumor Size.

As shown in **Figure 2**, among patients with N0 stage, there were no significant differences in TCSS among patients with different tumor sizes. However, for patients with N1a stage, tumor size had minimal impact on TCSS for tumors measuring 13mm or less. Patients with tumors measuring 11-13mm had significantly better TCSS than those with tumors measuring 14-16mm, and patients with tumors measuring 14-16mm had better survival than those with tumors measuring 17-20mm. Similarly, among patients with N1b stage, apart from patients with tumors measuring 11-13mm who had better survival than those with tumors measuring 14-16mm, different tumor sizes had little impact on survival prognosis. As shown in **Figure 3**, among patients with tumors measuring 13mm or less, there were no significant differences in TCSS among patients with lymph node stages of N0, N1a, and N1b. Only among patients with tumors measuring 14mm or larger, those with N1b lymph node stage had worse prognosis compared to patients with N1a stage.

### Survival benefits adjusted for IPTW

**Figure 4:**
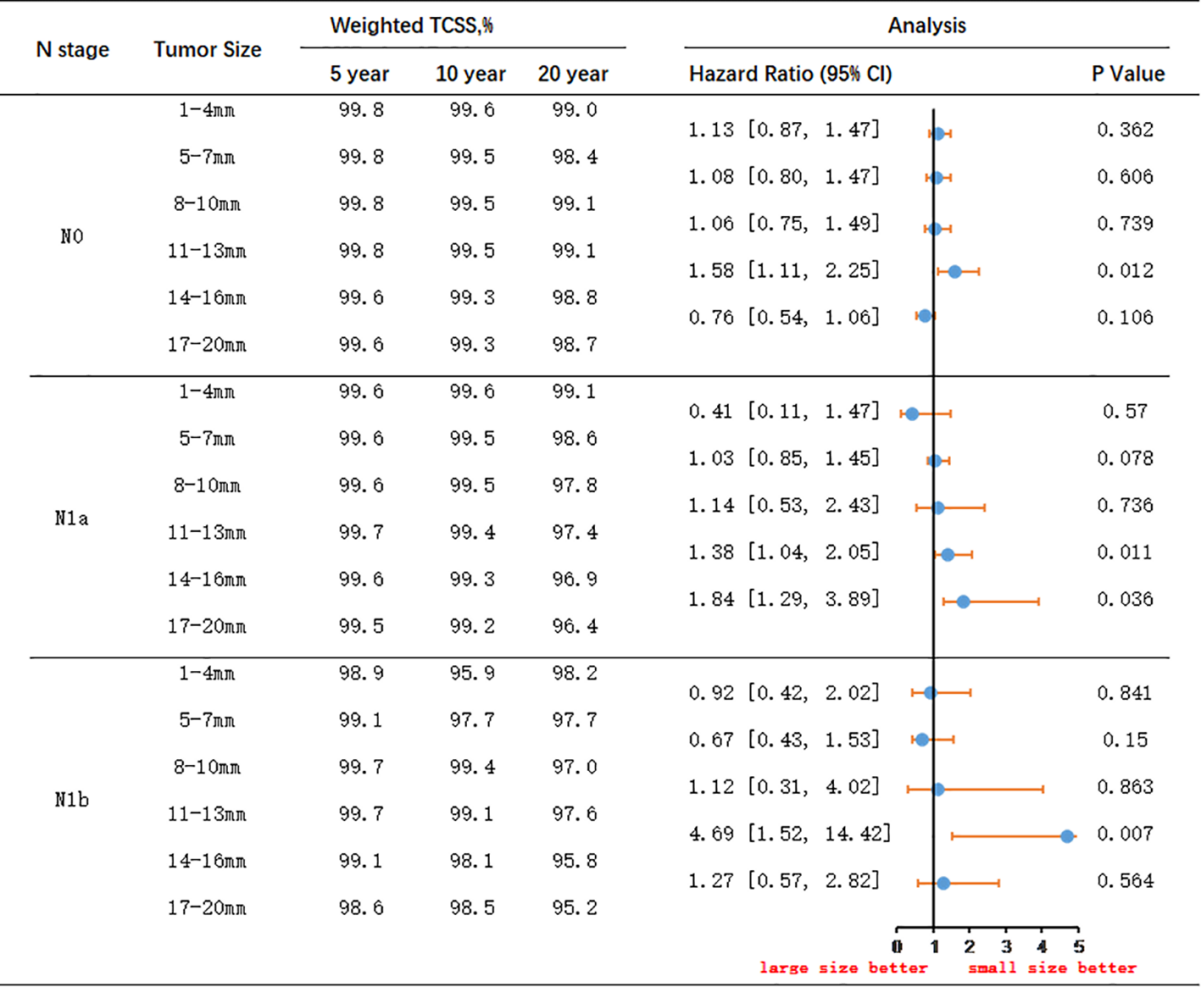
IPTW-Adjusted Hazard Ratio comparing Thyroid Cancer-Specific Survival Rates of Patients with Different Tumor Sizes: 1-4mm vs. 5-7mm, 5-7mm vs. 8-10mm, 8-10mm vs. 11-13mm, 11-13mm vs. 14-16mm, 14-16mm vs. 17-20mm, Stratified by Lymph Node Status. TCSS, thyroid cancer–specific survival.

**Figure 5:**
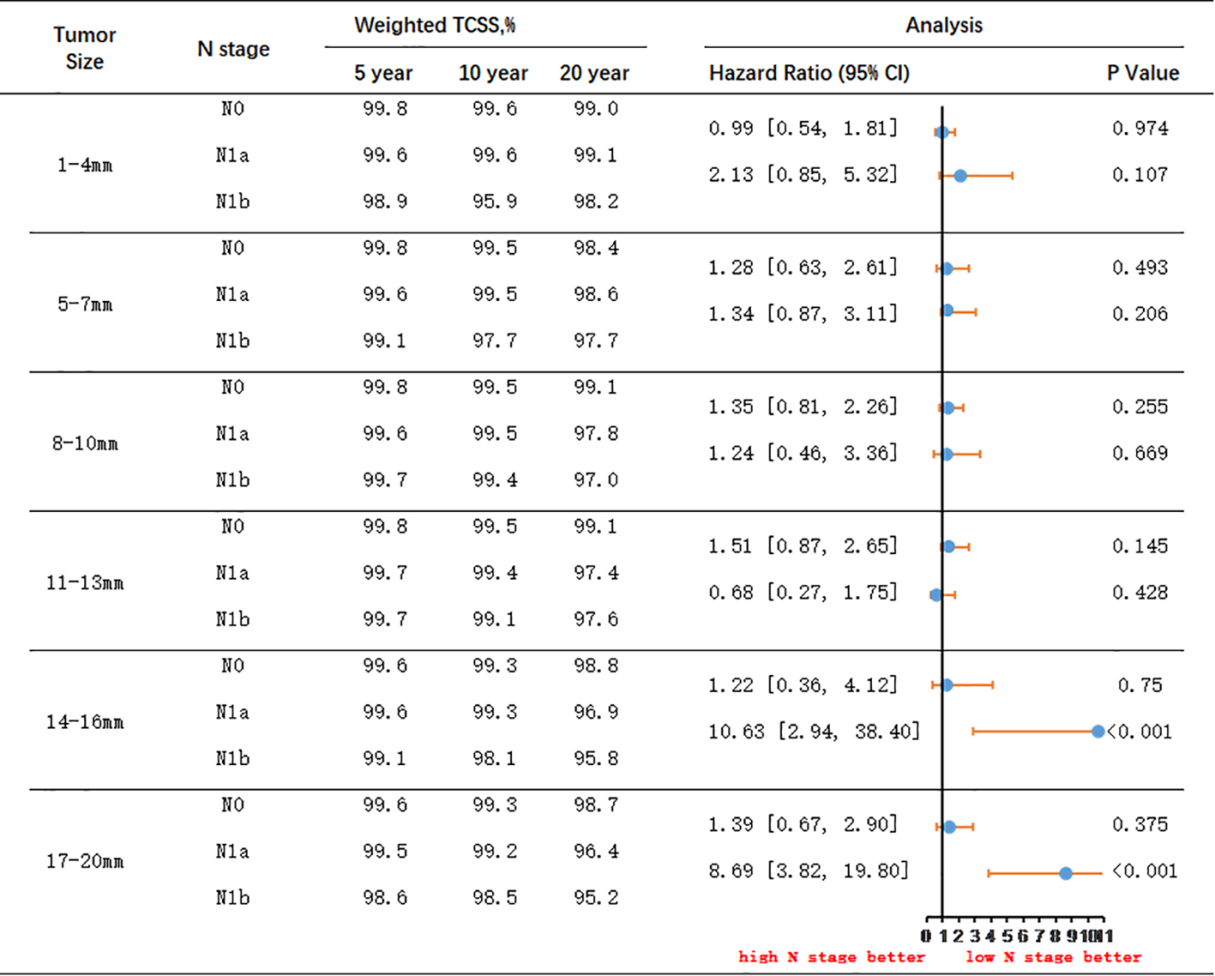
IPTW-Adjusted Hazard Ratio Comparing Thyroid Cancer-Specific Survival Rates of Patients with Different Lymph Node Status: N0 to N1a and N1a to N1b, Stratified by Tumor Size. TCSS, thyroid cancer–specific survival.

**Figures 4** and **5** present the outcomes of a multivariable survival analysis adjusted using IPTW for different tumor sizes and N stages. In patients with N0 stage, a significant difference in survival was found only between the groups with tumor sizes of 11-13mm and 14-16mm. Patients with tumor sizes of 11-13mm had a significantly better prognosis compared to those with sizes of 14-16mm (HR 1.58, 95% CI 1.11-2.25). The 5-year TCSS rate for patients with tumor sizes of 11-13mm was 99.8%, with 10-year and 20-year survival rates of 99.5% and 99.1%, respectively. For patients with tumor sizes of 14-16mm, the 5-year survival rate was 99.6%, the 10-year survival rate was 99.3%, and the 20-year survival rate was 98.8%. The absolute survival differences between the two groups were 0.2% at 5 years, 0.2% at 10 years, and 0.3% at 20 years.

Among patients with N1a stage, significant differences in survival were observed between tumor sizes of 11-13mm and 14-16mm, as well as between 14-16mm and 17-20mm. Patients with tumor sizes of 11-13mm had a significantly better prognosis than those with 14-16mm tumors (HR 1.38, 95% CI 1.04-2.05). For patients with tumor sizes of 11-13mm, the 5-year TCSS rate was 99.7%, the 10-year survival rate was 99.4%, and the 20-year survival rate was 97.4%. Conversely, for patients with tumor sizes of 14-16mm, the 5-year survival rate was 99.6%, the 10-year survival rate was 99.3%, and the 20-year survival rate was 96.9%. The absolute survival differences between the two groups were 0.1% at 5 years, 0.1% at 10 years, and 0.5% at 20 years. Additionally, significant differences in survival were observed between patients with tumor sizes of 14-16mm and 17-20mm. Patients with tumors sized 14-16mm had a significantly better prognosis than those with tumors sized 17-20mm (HR 1.84, 95% CI 1.29-3.89). For patients with tumors sized 14-16mm, the 5-year TCSS rate was 99.6%, the 10-year survival rate was 99.3%, and the 20-year survival rate was 96.9%. On the other hand, for patients with tumors sized 17-20mm, the 5-year survival rate was 99.5%, the 10-year survival rate was 99.2%, and the 20-year survival rate was 96.4%. The absolute survival differences between the two groups were 0.1% at 5 years, 0.1% at 10 years, and 0.5% at 20 years.

In patients with N1b stage, significant differences in survival were observed between patients with tumor sizes of 11-13mm and 14-16mm. Patients with tumors sized 11-13mm had a significantly better prognosis than those with tumors sized 14-16mm (HR 4.69, 95% CI 1.52-14.42). For patients with tumors sized 11-13mm, the 5-year TCSS rate was 99.7%, the 10-year survival rate was 99.1%, and the 20-year survival rate was 97.6%. Conversely, for patients with tumors sized 14-16mm, the 5-year survival rate was 99.1%, the 10-year survival rate was 98.1%, and the 20-year survival rate was 95.8%. The absolute survival differences between the two groups were 0.6% at 5 years, 1.0% at 10 years, and 1.8% at 20 years.

Among patients with tumor size less than 13mm, there were no significant differences in survival prognosis between those with N0 and N1a stage, or between N1a and N1b stage. However, in patients with tumor size between 14-16mm, although there was no significant difference between N0 and N1a stage, there was a significant difference between N1a and N1b stage. Patients with N1a stage had significantly better TCSS prognosis compared to those with N1b stage (HR 10.63, 95% CI 2.94-38.40). For patients with tumor size between 14-16mm and N1a stage, the 5-year survival rate was 99.6%, the 10-year survival rate was 99.3%, and the 20-year survival rate was 96.9%. Conversely, for patients with N1b stage, the 5-year survival rate was 99.1%, the 10-year survival rate was 98.1%, and the 20-year survival rate was 95.8%. The absolute survival differences between the two groups were 0.5% at 5 years, 1.2% at 10 years, and 1.1% at 20 years.

In patients with tumor size between 17-20mm, there were no significant differences in survival prognosis between those with N0 and N1a stage. However, patients with N1a stage had a significantly better prognosis compared to those with N1b stage (HR 8.69, 95% CI 3.82-19.80). For N1a stage patients with tumor size between 17-20mm, the 5-year survival rate was 99.5%, the 10-year survival rate was 99.2%, and the 20-year survival rate was 96.4%. Conversely, for N1b stage patients, the 5-year survival rate was 98.6%, the 10-year survival rate was 98.5%, and the 20-year survival rate was 95.2%. The absolute survival differences between the two groups were 0.9% at 5 years, 0.7% at 10 years, and 1.2% at 20 years.

To assess the reliability of our results, sensitivity analyses were conducted, yielding similar findings. These analyses employed the proportional subdistribution hazards model and utilized random survival forest methodology after multiple imputation of missing data.

## Discussion

Recent research has highlighted concerns regarding the detection and treatment of small PTC, as it does not necessarily lead to reduced mortality rates. This issue of overdiagnosis and overtreatment in low-risk PTC cases has raised questions about patient quality of life and public health management. To address overdiagnosis, various guidelines and recommendations have been put forth. For instance, the U.S. Preventive Services Task Force advised against screening for thyroid cancer in asymptomatic adults using neck palpation or ultrasound (19). Similarly, the American Thyroid Association and the Japan Society of Ultrasonics in Medicine recommended observation rather than fine-needle aspiration for small thyroid nodules (10, 20).

While AS is an excellent strategy, it may not be suitable for all patients with PTMC, as some cases may demonstrate aggressive behavior. Identifying low-risk PTMCs that may demonstrate more aggressive behavior is important. Factors such as distant metastasis at diagnosis (very rare), vocal cord paralysis due to invasion of the recurrent laryngeal nerve, or highly malignant tumors based on cytology are generally considered unsuitable for AS (21). However, there is debate regarding the suitability of tumors located near critical structures such as trachea or recurrent laryngeal nerve. Recent studies have indicated that tumors smaller than 9mm, regardless of location, do not typically invade critical structures (22, 23).

Patients undergoing AS undergo regular ultrasound monitoring, with surgery recommended if there is an increase of 3 mm or more in the maximum diameter of PTMC or the detection of lymph node metastasis (21). In this study, patients were stratified into different risk groups based on tumor size and N stage according to criteria for disease progression, aiming to compare the survival prognosis of patients with varying levels of risk. The study findings indicate that patients in N1a stage have similar prognosis as those in N0 stage. Despite the difficulty of accurately identifying preoperative central compartment lymph node metastasis using ultrasound, delaying surgery does not significantly impact patient outcomes even in the presence of occult central compartment lymph node metastasis.

Usually, the indication for AS is limited to T1aN0M0 PTC, excluding T1bN0M0. However, studies have shown similar survival rates between T1a and T1b patients who underwent surgery (24). Limited research has focused on long-term AS for T1b tumors. The findings of this study reveal that patients with tumors smaller than 13mm had similar prognoses regardless of lymph node status. Thus, delaying surgery did not significantly affect outcomes for T1a patients, even if lateral cervical lymph node metastasis occurred or the tumor size increases to 13mm during AS. Similar results were observed in data from Kuma Hospital and the Cancer Institute Hospital, where patients with slight progression of PTMC who underwent rescue surgery did not experience life-threatening recurrence or death from thyroid carcinoma (21). In patients with T1b stage and a tumor size larger than 13mm, lateral cervical lymph node metastasis had a notable impact on TCSS, although the absolute survival difference over 20 years was only about 1%. This finding suggests that for patients with poorer baseline health or shorter life expectancy, AS can be considered as an alternative to immediate surgical treatment.

Age plays a significant role in the management of differentiated thyroid cancer, and it is noteworthy that age is considered a prognostic factor for this particular malignancy (25–28). Studies from Japan have indicated that younger age is associated with tumor growth during AS (7, 29). The findings of the present study were similar in that younger patients present with noticeably larger tumor sizes and a higher number of lymph node metastases, which progressively increase with advancing age. Furthermore, an additional study revealed that the estimated lifetime probabilities of disease progression for patients in their 20s, 30s, 40s, 50s, 60s, and 70s were 48.6%, 25.3%, 20.9%, 10.3%, 8.2%, and 3.5%, respectively (29). These findings suggest that approximately half of the patients in their 20s will require surgery due to disease progression, while the other half may not need surgery throughout their lifetime. Importantly, patients who underwent rescue surgery after disease progression did not experience life-threatening recurrence or death from thyroid carcinoma. Therefore, despite the higher risk of disease progression in young patients with PTMC, AS remains a viable option.

As mentioned above, there is an inverse relationship between age and the progression of low-risk PTMC. Our research confirms that in patients over the age of 30, the average tumor size gradually decreases. This could be attributed to two reasons. Firstly, PTC typically grow slowly, especially in elderly patients, where the tumor size remains stable or changes minimally. Secondly, older patients may develop new smaller tumors, thereby reducing the average tumor size. However, it is important to note that this does not mean that older patients do not require regular monitoring, as advanced age is an important factor associated with poor prognosis (30). Although rare, if PTMC progresses without early detection in older patients, it can pose a life-threatening situation. Currently, there is no available evidence indicating when and at what age AS can be discontinued.

Continuing AS throughout one’s life is a reasonable option. Even after surgery, regular postoperative follow-up and thyroid hormone administration may still be necessary. Oda et al. conducted a study comparing the 10-year medical costs between immediate surgery and AS. Their findings within the Japanese medical system revealed that the costs in the immediate surgery group were approximately 4.1 times higher than those in the AS group (31). Similarly, a report from Hong Kong showed that the medical costs of the AS group remained lower for 16 years after treatment initiation, and the cost-effectiveness further improved when compared to the immediate surgery group (32).

Furthermore, AS can prevent surgery-related complications and adverse events, leading to further reduction in healthcare costs. However, it may also induce anxiety regarding disease progression. Several studies have indicated that patients with stage T1N0M0 PTMC who did not undergo surgery had significantly lower overall survival rates compared to those who did undergo surgery (33). This difference may be partly attributed to excessive psychological stress and poor medical compliance. Therefore, it is crucial to alleviate the psychological burden of PTMC patients during AS, encourage adherence to standardized AS protocols, ensure good medical compliance, and minimize the impact of comorbidities. Providing appropriate medical education to patients can help reduce cancer-related stress and surveillance-related stress, enhance patient compliance with AS protocols, and improve follow-up rates (6).

Our study has several limitations. Firstly, the data were retrospectively obtained solely from the SEER database, lacking prospective data on patients with low-risk PTC. Additionally, our analysis focused only on the prognosis of low-risk patients based on TCSS and did not incorporate information on recurrence, which may have resulted in an overestimation of the results. Furthermore, we did not explore the impact of recurrence risk and treatment approaches on the outcomes. For patients with small PTC and N1b or N1a lymph node metastasis, selecting an appropriate surgical extent is of paramount importance. Lobectomy alone may not completely eradicate lymph node metastasis, thereby increasing the risk of recurrence. The risk of recurrence plays a pivotal role in determining the necessity of total thyroidectomy following lobectomy or when considering adjuvant RAI treatment. Another limitation is the lack of evaluation or consideration of family history, vascular invasion, other histologic findings, preoperative ultrasonographic data, and molecular mutations (such as BRAF, RAS, and TERT mutations) in our study. Furthermore, as the SEER database is based on US data, the generalizability of the findings to a global context may be limited.

## Conclusion

Apart from young patients, the likelihood of disease progression during the AS of low-risk PTC is relatively low. Furthermore, even if the tumor progresses, such as an increase in size (but with a diameter ≤13mm) or the presence of central neck lymph node metastasis, delaying surgery has no significant impact on the TCSS. When the tumor size exceeds 13mm or is accompanied by lateral neck lymph node metastasis, the TCSS of the patient is affected, but the absolute impact on TCSS rate is minimal. Therefore, even if the diameter of PTC exceeds 13mm, adopting AS remains a feasible strategy in cases where patients have a shorter life expectancy or poor baseline health condition.

## Data availability statement

The raw data supporting the conclusions of this article will be made available by the authors, without undue reservation.

## Author contributions

Conception and design: WD, DY, JZ, GR. Administrative support: YL, ZL. Collection and assembly of data: WD, YL, ZL. Data analysis and interpretation: WD, DY, JZ. Manuscript writing: All authors. Final approval of manuscript: All authors. All authors contributed to the article and approved the submitted version.
